# The Impact of the Tumor Microenvironment on the Properties of Glioma Stem-Like Cells

**DOI:** 10.3389/fonc.2017.00143

**Published:** 2017-07-10

**Authors:** Alessandra Audia, Siobhan Conroy, Rainer Glass, Krishna P. L. Bhat

**Affiliations:** ^1^Department of Translational Molecular Pathology, University of Texas, M.D. Anderson Cancer Center, Houston, TX, United States; ^2^Department of Pathology and Medical Biology, University Medical Center, Groningen, Netherlands; ^3^Neurosurgical Research, Department of Neurosurgery, University Hospital, LMU Munich, Munich, Germany; ^4^German Cancer Consortium (DKTK) partner site Munich and German Cancer Research Center (DKFZ), Heidelberg, Germany; ^5^Department of Neurosurgery, University of Texas, M.D. Anderson Cancer Center, Houston, TX, United States

**Keywords:** glioma stem-like cells, glioblastoma, microenvironment, transdifferentiation, therapy resistance

## Abstract

Glioblastoma is the most common and highly malignant primary brain tumor, and patients affected with this disease exhibit a uniformly dismal prognosis. Glioma stem-like cells (GSCs) are a subset of cells within the bulk tumor that possess self-renewal and multi-lineage differentiation properties similar to somatic stem cells. These cells also are at the apex of the cellular hierarchy and cause tumor initiation and expansion after chemo-radiation. These traits make them an attractive target for therapeutic development. Because GSCs are dependent on the brain microenvironment for their growth, and because non-tumorigenic cell types in the microenvironment can influence GSC phenotypes and treatment response, a better understanding of these cell types is needed. In this review, we provide a focused overview of the contributions from the microenvironment to GSC homing, maintenance, phenotypic plasticity, and tumor initiation. The interaction of GSCs with the vascular compartment, mesenchymal stem cells, immune system, and normal brain cell types are discussed. Studies that provide mechanistic insight into each of these GSC–microenvironment interactions are warranted in the future.

## Introduction

Our view of cancer has changed ever since the discovery of cancer stem cells (CSCs) (1). CSCs are a proportion of cells within the bulk tumor and are similar to normal stem cells in their ability to self-renew and differentiate into downstream lineages. These cells exhibit tumor-initiating potential compare to the non-CSCs counterpart or differentiated progeny (2). CSCs have revolutionized our understanding of tumor biology and have had a strong impact on strategies for tumor treatment. However, the criteria to clearly define CSCs are well established in some tumors (for, e.g., leukemias), but are less clear for primary brain tumors such as glioblastomas (GBMs) (3–5). Glioma stem-like cells (GSCs) in GBM were first described about decade and a half ago (6–8). Since then, many studies have not only confirmed their existence but also identified additional attributes to these cell types including contribution to therapy resistance (9, 10). Numerous alternative terminologies have been used for GSCs including glioma-initiating cells, glioma stem cells, and glioma tumor propagating cells. The inherent difficulties to up-front identify stem-like cells in GBM has stirred a long (and ongoing) debate about the nature, pathological impact, and therapeutic value of GSCs (11, 12). Furthermore, the population dynamics of these cells with cell fate conversion and retention of stem-like properties perhaps by de-differentiation further complicates matters. In order to treat this highly malignant disease, a better understanding of these cells is necessary. Furthermore, due to the therapeutic resistance of GSCs, understanding not only the inherent resistance mechanisms but also the contribution of the surrounding microenvironment could be considered as an interesting alternate approach. Interactions of GSCs with the microenvironment not only contributes to maintenance of the stem-like state of the cells that eventually leads to clonal expansion but also acquires aggressive traits including migration, invasion, and therapy resistance. In this review, we will discuss the cell types present in the tumor microenvironment and the type of interactions with the microenvironment that influence expansion of GSCs. We will focus primarily on studies that have examined GSCs in the context of the microenvironment, but studies that by definition have not examined the stem cell component in tumor cells have also been included in some cases where there is insufficient literature on GSC interaction with a specific cell type.

## CSCs in GBM

For a better understanding of the microenvironment, it is imperative to first briefly comprehend the history and the controversies underlying the fundamental definition of CSCs in GBM. Several detailed reviews exclusively focused on GSCs have already been published (11, 13). Here, we present a general opinion on the current state of the GSCs. A robust definition for a GSC may follow established biological criteria that are generally applied to all stem cells (including non-tumorigenic stem cells), which is, the ability to clonally expand and to give rise to more differentiated progeny (14). For GBMs, these criteria were first applied by Peter Dirks (15), thus identifying GSCs, which were also endowed with tumor-initiating capacity. This seminal study identified a marker (prominin, CD133) to purify GSCs and to test stemness properties. Using the tumor-initiating capacity as a benchmark for CSC has been tricky since this feature is assayed by tumor implantation experiments using decreasing numbers of GSCs to monitor tumor take in cells positive or negative for cell surface markers (15). However, not all studies strictly adhere to these benchmarks. Ever since this first publication, the field of GSCs has exploded with its share of great advances and controversies. First, using CD133 as a standalone marker has not been a successful and reproducible strategy. This, in part, could be attributed to the rapid alteration of stem cell (CD133^+^) populations in extended *in vitro* cultures. However, CD133 negative populations from freshly dissociated tumors have been also shown to form tumors at similar rates as CD133^+^ cells and additional markers for defining GSCs have been proposed (11). Also, it is noteworthy that CD133 undergoes N-glycosylation upon cellular differentiation, whereas and many studies have been examining changes in mRNA expression which bears no relevance to stemness (16). More importantly, GSCs from a single GBM can express multiple markers and tumor-initiating potential of each of these populations can vary (17). Second, the proportion of GSCs in a GBM may vary substantially between GBM of different individuals (18) prohibiting a generalized method to quantify, compare, and thereby standardize the aggressiveness of GSCs from different donors in xenograft transplantation assays. Diversity in GSC subtypes is likely associated with the established intratumoral heterogeneity of GBM (19), which is propelled by clonal evolution generating different tumor areas (within the same tumor) that are predominated by progeny from different tumor cell clones (20, 21). The coexistence of evolutionary evolved and genetically distinct tumor subfields in one GBM is a relatively recent observation and was previously not routinely taken into account in studies using GSCs. Third, the interest in research in GSC biology is high, since, apart from a potential role in tumor initiation and relapse (22), GSCs have been attributed with other clinical problems such as resistance to radiation (9, 10) or chemotherapy (23, 24). These are all important points adding to the pathological potential of GSCs and can support the notion that GSCs are a major cell entity that forms recurrent tumors after multi-modal treatment. However, therapeutic resistance cannot serve as a defining criterion of GSC as there is ample heterogeneity also in therapy response of GSCs (10, 25) and high therapeutic resistance can as well be detected in more differentiated GBM cells [non-GSCs (26)]. Despite these controversies and differences, GSCs are central in our understanding of GBM biology.

## The Effect of the Vasculature on GSC Properties

### Endothelial Cells

The major neural stem cell pools in the adult mammalian brain are confined to the subventricular and subgranular zones, each with a defined set of surrounding cells that protect their stem-like state (27). The brain tumor stem-like cells were first reported to preferentially reside in the perivascular niche (28). Increased numbers of endothelial cells expanded the fraction of stem-like cells, and conversely *in vivo* blood vessel depletion through anti-angiogenic agents considerably slowed tumor growth and decreased the count of self-renewing and multipotent cells (28). The site where stem-like cells generally reside is considered relatively hypoxic, an environmental cue that is transcriptionally converted by cells into stabilization of hypoxia-inducible factor (HIF-1α). In glioma cells, both HIF-1α and HIF-2α enhance glioma sphere formation and cell proliferation, induce stemness, and increase tumor initiation ability (29, 30). Besides affecting the GSCs themselves, the stabilization of HIF-1α profoundly affects the vascular compartment through the induced secretion of VEGFA (9), which creates a gradient for the developed vessels, coordinating tip cell selection and stalk elongation in endothelial cells, and promoting endothelial sprouting or angiogenesis (31).

In addition to VEGFA, other angiogenic signaling molecules such as endothelial cell-bound ligands DLL4 and Jagged-1 can bind the Notch receptors expressed on GSCs. Blockade of Notch signaling in xenograft GBMs through γ-secretase inhibitors inhibits tumor growth establishing a role for Notch signaling for GSC maintenance and tumor growth (32, 33). Adjacent localization of Nestin^+^ and Notch^+^ tumor cells and Notch ligand expressing endothelial cells was observed in primary GBMs (34). Co-culturing of endothelial cells with GBM neurospheres enhanced cell growth, which could be inhibited through knockdown of these endothelial ligands. Nitric oxide (NO) is another signaling molecule that is produced by endothelial cells. NO is produced from the substrate l-arginine through a family of NO synthases, of which the endothelial isoform is denominated eNOS. In GBMs, eNOS expression is elevated and correlated with increased tumor growth (35, 36). PDGF-driven eNOS^−/−^ glioma bearing mice prolonged survival *in vivo* and decreased Notch signaling, hence establishing the GSC beneficial effects of Notch signaling described above through another route. Conservation of this pathway in *PDGFR*-amplified human glioma specimens was also shown.

In addition to the bi-directional interaction between GSCs and endothelial cells, GSCs directly impact vasculature through transdifferentiation mechanisms. GSCs have been shown to generate functional vasculature, acquire endothelial-like properties both *in vitro* and *in vivo*, and ablation of the transdifferentiation process slowed tumor growth (37, 38). The acquisition of the CD105 endothelial marker expression was shown to be controlled by Notch signaling and endothelial cells were shown to harbor genomic aberrations similar to those observed in the tumor cells. A recent study showed glioma cells transdifferentiating into endothelial cells in a p53-inactivated/AKT-driven glioma model under epigenetic control of the WNT signaling pathway (39). This study reported that the endothelial transdifferentiation was specifically observed at the invasive site of the tumor in contrast to earlier studies that showed the presence of endothelial cells from human origin in the core of the tumors. However, follow-up clinical studies could not confirm the widespread *EGFR* amplified CD34^+^ endothelial cells in gliomas (40), and hence transdifferentiation, while important, may be a rare event in the evolution of gliomas.

### Pericytes

Pericytes are vascular smooth muscle cells that provide support, maintain vascular integrity, stabilize the vessels, and prevent vascular leakiness (41). While the exact contribution of pericytes to GSC self-renewal and tumor initiation is still unknown, GSCs were recently shown to transdifferentiate into pericytes (42). Using lineage specific reporters, it was demonstrated that pericytes were GSC derived. GSCs were recruited by SDF-1 signals from endothelial cells and were subsequently transdifferentiated into pericytes on-site under the influence of TGF-β. The contribution of GSCs to the pericyte pool was estimated to be substantial, with an average of 78% of pericytes carrying the tumor marker. Once again, this notion has been challenged in other pericyte reporter mouse strains that were used as glioma models. In one study, researchers used transgenic animals expressing GFP under control of the pericyte-associated signaling molecule G protein signaling 5 (RGS5-GFP) (43) and did not detect any contribution of syngeneic glioma cells (GL261 cells) to perivascular structures. Likewise, when implanting a mouse glioma cell line into a dual fluorescence pericyte reporter animal model (44), the pericytes appeared to be derived from the host tissue. The mouse glioma cell lines/models used in these two studies may not truly represent GSCs, which can explain why there is no strong transdifferentiation of tumor cells into pericytes. However, these reports suggest that transdifferentiation of GSCs is at least no prerequisite for the formation of new pericytes in GBM. Further studies using models that mimic the actual physiological tumor more closely are warranted in the case of transdifferentiation studies.

### Normal Brain–GSC Interactions

The adult brain is mainly composed of astrocytes, neurons, and oligodendrocytes. The role of these cells in tumor initiation and maintenance are relatively understudied, but the few studies that are available indicate that they do have an important role in GBM biology (45, 46). Around 50% of the brain cells are astrocytes, which serve a multitude of functions in homeostatic maintenance (47). In response to injury or surgery, astrocytes can become activated and are often termed reactive astrocytes (48). GSCs were able to decrease p53 expression in the surrounding reactive astrocytes, thereby making them acquire a tumor-permissive or even promoting phenotype (49). Astrocytes surrounding a xenograft express higher levels of Connexin43 that facilitates glioma invasion (50). Astrocyte injury also caused transcriptome and secretome alterations *in vitro*, and enhanced GBM cell proliferation and invasion (51). The role of neurons and oligodendrocytes on GSC biology are less known, but a recent report showed that neuronal activity-induced secretion of neuroligin-3 promotes gliomagenesis (52). Although these effects were not studied using GSCs, further studies are required to confirm and elaborate on the findings of the few available reports that indicate that normal brain cell types play an important role in GSC functions.

### The Immune Microenvironment Surrounding GSCs

With the advent of immunotherapy, the past few years has seen an explosion of studies describing the immune system and its critical role in cancer pathogenesis (53, 54). However, the immune regulation of brain tumors, in the context of GSCs, is not fully established. It should be noted that one major caveat in studies involving GSCs is that tumor initiation and evolution is studied in xenograft bearing mice that have greatly reduced number of T cells, and therefore, studies involving GSCs and the immune system can never be physiologically complete.

Although for decades the brain has been considered as immune privileged, owing to an intact blood–brain barrier and the “absence” of lymphatic drainage system, recent discoveries prove the existence of a direct communication between the CNS and the immune system. A variety of immune cell types including microglia and macrophages, T lymphocytes, and dendritic cells (DCs) are found in the brain and play a role in immune surveillance (55). The activation of the immune system or its suppression in the GBM microenvironment depends on cell type/function and on the presence/absence of immune signals in the local environment.

### T Lymphocytes

Some earlier studies describing the role of immune cells in GSCs have been generally shown to silence the immune response, escape immune surveillance, for instance, with an ineffective tumor antigen presentation, or release and recruitment of on-site immune suppressive factors (such as TGF-β) and immune suppressive cells (such as immunosuppressive B-cells and myeloid cells) (55). Studies have shown that GSCs can mimic antigen-presenting cells in their expression of major histocompatibility complex I (MHC I). GSCs can either regulate the level of expression of the MHC I complex (56) or express the inhibitory co-stimulating molecule B7 homolog 1 (also known as programmed death ligand 1) and lack the expression of the activating co-stimulating molecules CD40, CD80, and CD86. Without MHC I expression or co-stimulating factors, cancer cells fail as antigen presenting cells or induce T cells to anergy following antigen presentation, rendering them incapable of being activated. PD-L1 has been shown being upregulated in the GBM microenvironment and it seems to be more associated with the mesenchymal subtype (57, 58).

In addition to expression of co-inhibitory molecules, alternative mechanisms of T cell inhibition exist. Regulatory T cells (Tregs) are a form of T cells that are immunosuppressive and generally inhibit the expansion of effector T cells. This is attributed to constitutive activation of cytotoxic T-lymphocyte-associated protein 4 (CTLA-4), which contributes to their ability to suppress the immune system (59). The ligand CD95 (Fas/apoptosis antigen 1) is expressed on GSCs and induce apoptosis of Tregs and reduce the number of infiltrating T cells in the tumor microenvironment (60, 61). Similarly, CTLA-4 present on activated Tregs can bind CD28 and induce T cell anergy (62). An interesting crosstalk mechanism between GSCs and T cells is the secretion of galectin-3 triggered apoptosis in both naïve and activated T cells promoting the expansion of the CSCs and therefore their immune suppressive role (63, 64). Alternatively, GSCs cause immunosuppression in the glioma microenvironment by activating the STAT3 pathway and by increasing the number of Tregs (65). On these lines, it has been shown that GSCs secrete more TGF-β than their differentiated counterparts (66). TGF-β is involved in the down-modulation of MHC II expression and subsequent antigen processing and in the expansion of immune suppressive Treg cell population and perhaps is one additional mechanism by which GSCs modulates Tregs (67). Once again, it is noteworthy that owing to the heterogeneity, not all GBMs show a significant infiltration of Tregs in the microenvironment, suggesting heterogeneity also in the immune suppressive mechanisms. This emphasizes the necessity to study GSC interaction with T cells at the single cell level as well as distinguishing them according to their subtype and mutation profiles. In order to do so, novel mouse models are required that could better represent the heterogeneity of this tumor *in vivo*.

### Microglia and Macrophages

In addition to secreting immune suppressive cytokines, GSCs are capable of recruiting or modulating immune cells with tumor supportive phenotype. GBMs are characterized by a very high infiltration of macrophages, phagocytic cells that engulf cellular debris and foreign substances. Microglia, are resident macrophages that present the first line of defense in the CNS. Both microglia and infiltrating macrophages (myeloid cells) can represent between 13 and 43% of the tumor mass in different animal models for GBM (68) and in freshly isolated GBM biopsies a myeloid cell content of approximately 8% (69) was detected in FACS analyses, while in immunohistochemical studies, often a myeloid cell content ranging from 20 to 50% of the total cell mass is reported (70), which we (according to our experience) consider as representative.

In classical immunological experiments, investigating, e.g., the response of myeloid cells to toll-like receptor agonist (like lipopolysaccharides) or to chronic inflammatory stimuli, myeloid cells are often grouped into two different immune phenotypes named M2 (which is representative for the chronic inflammatory state) and M1 (which includes all classical pattern of inflammation) (71). In many different peripheral cancers, the attribute of an M2-shifted immune response of tumor-associated macrophages (TAMs) indicates a more tumor-supportive function that is associated with a myeloid cell signaling pattern that is highly reminiscent of the chronically activated state myeloid cells (71). The phagocytic role of TAMs (or glioma-associated macrophages/microglia) has been controversial. While one study showed a lack of phagocytic activity of these cell types (72), and their secreted cytokines, such us IL-6 and IL-10, can promote cancer cell proliferation (73), other studies have shown that factors secreted by glioma cells can promote phagocytosis, and proliferation of microglial cells (74). Unlike their more differentiated progeny, GSCs show an increased capacity of active chemo-attraction and recruitment of macrophages *in vitro* through the secretion of cytokines. These include colony-stimulating factor-1 (CSF-1), C-C motif ligand-2 (CCL-2), and macrophage inhibitory cytokine 1, factors enriched in GSC-conditioned media. Moreover, the secretion of CSF-1 and CCL-2 by the GSCs resulted in a polarization of the macrophages toward the M2 immune suppressive phenotype. Other CSC-secreted factors include IL-10 and TGF-β, which also suppress tumor-associated microglia/macrophage function and generate a more immunosuppressive (M2) phenotype (75). TAMs in GBM have been shown to be recruited by periostin, a protein preferentially expressed by GSCs (76). Periostin functions as a potent chemo-attractant of monocyte-derived macrophages from the blood to the tumor microenvironment and maintain the M2 immune suppressive phenotype to promote tumor growth. In fact, the disruption of periostin *in vivo* shows a reduction in the recruitment of tumor-supportive TAMs (M2 subtype), inhibition of tumor growth, apoptosis of GSCs, and increase of survival in a xenograft mouse model. Another molecule with crucial role in this transition is SPP1 (osteopontin). SPP1 is a secreted protein that shares similarities in features and mechanism of action with Periostin and contributes to the maintenance of an immune suppressive environment. It has been shown, in fact, to be highly expressed in M2 polarized macrophages and GBM-infiltrating CD14^+^ cells compared to matched blood monocytes and brain microglia (77).

It is important to note that gene expression studies of TAMs from GBM have shown that such a simplified classification is not feasible in brain tumors, which likely harbor a spectrum of differently polarized myeloid cells that (as a net effect) culminates in a GBM-promoting role of TAMs (78).

### Myeloid-Derived Suppressor Cells (MDSCs), Neutrophils, Natural Killer (NK) Cells, and DCs

Myeloid-derived suppressor cells are immature cells of myeloid origin that possess T cell suppressive properties. Patients with GBM have increased counts of MDSCs compared to healthy subjects (79). A recent detailed analysis of the phenotype of MDSCs and transcriptome of the different population of myeloid cells in GBM patient samples revealed the abundance of the MDSCs and microglia in the microenvironment highlighting the importance of the innate immune components (78). These data showed a higher infiltration of MDSCs in the microenvironment and detailed their role in the determination of the immune suppressive phenotype. MDSCs were elevated in many GBM patients in association with neutrophils and the number of neutrophils correlates with glioma grade and a negative prognosticator for survival (78). This is again consistent with demonstration of neutrophil infiltration in high grade gliomas in previous studies (80). The role of MDSCs in GSC biology was never studied until two recent reports. One study showed that GSCs reside in proximity to and attract MDSCs by secreting macrophage migration inhibitory factor (MIF) (81). MDSCs suppressed immune rejection and caused expansion of GSCs, which could be reversed by inhibition of MIF. In another study, the authors showed that GSCs secrete exosomes that regulate monocyte maturation and MDSC formation that in turn suppresses T-cell response (82).

Natural killer cells are cytotoxic lymphocytes and belong to the innate immune system. They cause host rejection and killing of tumors and microbial infections by cytokine release selectively against cells that lack the MHC class I, thereby protecting normal host cells from attack (since all normal cells express this antigen) (83). In recent years, NK cells have been shown to play a role in GBM-mediated immune suppression. IDH-mutant GSCs have been shown to have a significantly lower expression of NKG2D ligand compared to the IDH-wt cells, rendering the IDH-mutant GSCs resistant to NK cell-mediated lysis. Decitabine-mediated hypomethylation upregulates the expression of NKG2D ligand restoring the NK-mediated lysis of IDH-mut GSCs (84). The novelty of this study resides not only in the description of a mechanism of immune suppression mediated by a single mutation but also show an intrinsic altered mechanism of the innate immune system that might explain in part the failure of immunotherapy for IDH-mutant gliomas. Moreover, it has been shown that human GSCs express lower levels of the PD-1 ligand and therefore are more sensitive to the cytotoxicity of the IL-activated NK cells (85). Combining the negative immune regulation of the PD-1/B7H1 pathway (86) and the anti-GSCs effect of stimulated NK cells, the blockade of the PD-1/B7H1 pathway between NK and GSCs might interrupt immunosuppression and promote NK cells killing GSCs. It has been demonstrated, in fact, that inhibiting the PD-1/B7H1 pathway promotes the toxicity of NK cells against GSCs *in vitro* (87). In an intracranial GSC model, mice that received PD-1-inhibited NK cell treatment showed reduced tumor growth and survived longer without obvious body weight loss or distinct neurological deficits (87). These studies open a new avenue of investigation and possibility of combinatorial therapy against the innate and adaptive immune system in GBM (88).

Dendritic cells are antigen-presenting cells to T lymphocytes and play a major role in initiating and shaping the adaptive response. These cells represent the most potent and versatile way the immune system has evolved to present antigens and develop an immune response (89). DCs may play immune suppressive mechanisms through dysregulation of the antigen-presenting pathway or inducing exhaustion of the T cells (90–92). Unfortunately, studies involving GSC–DC interactions are limited to developing vaccine strategies and inducing a satisfactory T-cell-mediated immune response (93).

## Conclusion

Studies involving GSCs have dramatically changed our view of this disease, and we have obtained a wealth of fascinating insight into the cell biology of GBM. The field of CSCs has helped us understand cancer biology from the perspective of developmental biologist. Similarities have been drawn to the cell types and differentiation pathways of normal stem cells. The recent information of GBM heterogeneity retrospectively explains why conflicting data were initially obtained in the GSC research field. It is now well established that GSCs are a central (but not the exclusive) target for therapeutic approaches to treat GBM. This is not only due to the inherent plasticity of GSCs that leads to transdifferentiation but also to the overbearing influence of the microenvironment (as highlighted in this review) that alters their stem cell state, tumorigenic potential, and therapeutic resistance. It is our opinion that future studies must incorporate the microenvironmental aspects while studying GSC biology.

## Author Contributions

AA and SC wrote the basic contents of the manuscript. KB conceived the manuscript, provided input throughout the preparation, and edited for the accuracy of the contents along with RG.

## Conflict of Interest Statement

The authors declare that the research was conducted in the absence of any commercial or financial relationships that could be construed as a potential conflict of interest.
